# The Ochodaeidae of Argentina (Coleoptera, Scarabaeoidea)

**DOI:** 10.3897/zookeys.174.2668

**Published:** 2012-03-09

**Authors:** M.J. Paulsen, Federico C. Ocampo

**Affiliations:** 1 Systematics Research Collections, University of Nebraska State Museum, W436 Nebraska Hall, Lincoln, NE 68588-0514 USA; 2 Instituto de Investigaciones de las Zonas Áridas – Instituto de Ciencias Básicas, CCT-CONICET Mendoza. CC 507, 5500. Mendoza, Argentina

**Keywords:** Systematics, Ochodaeidae, *Parochodaeus*, Chaetocanthinae, Argentina, new species, new genus

## Abstract

The Ochodaeidae (Coleoptera: Scarabaeoidea) of Argentina are revised. Previously, two species of Ochodaeinae were known from the country, both in the genus *Parochodaeus* Nikolajev: *Parochodaeus campsognathus* (Arrow) and *Parochodaeus cornutus* (Ohaus). An additional 7 species of *Parochodaeus* from Argentina are described here as new. In addition, *Gauchodaeus patagonicus*, new genus and new species in the subfamilyChaetocanthinae, is described. This is the first record of the subfamily Chaetocanthinae in South America. Redescriptions, diagnoses, and maps are provided for each species. We also provide a key to genera and a key to species of *Parochodaeus* of Argentina. With this work, the number of ochodaeid species known from Argentina is increased from 2 to 10.

## Introduction

The small family Ochodaeidae includes around 100 species of scarabaeoid beetles that are found nearly worldwide. The family is most diverse in arid, sandy habitats. They are sometimes referred to as the ‘sand-loving scarab beetles’ (Carlson 2001), although some species are found in forests. They can be distinguished from most other scarabaeoids by the presence of a pectinate or crenulate mesotibial spur and the absence of a true ocular canthus dividing the eye (Scholtz et al. 1988). Care should be taken in evaluating the presence of a canthus, because in many species of ochodaeids the first antennomere is greatly enlarged with a posterior lobe that covers a portion of the eye and appears as a ‘false canthus’.

Scholtz et al. (1988) examined the systematics of the family and divided it into two subfamilies with a total of five tribes. The subfamily Ochodaeinae, with two tribes, is absent only from Australia. It is particularly well represented in Africa, Madagascar, and southwestern North America. In South America only the genus *Parochodaeus* Nikolajev is known (Paulsen 2007, Paulsen in press). Species of *Parochodaeus* are distributed in the New World from the Great Plains of the United States to central Argentina. Among the Ochodaeinae, species of *Parochodaeus* can be easily distinguished by their elytral locking mechanism consisting of acute elytral apices and bituberculate propygidium (Nikolajev 1995; Paulsen 2007). Currently 17 species are known (Paulsen 2007; Paulsen 2011).

The second subfamily, Chaetocanthinae, is predominantly distributed in southern Africa, with three genera and seven species in total in that region. Also included in the subfamily is the monotypic genus *Pseudochodaeus* Carlson and Ritcher from western North America. Previously, no species were known to occur in South America. We independently discovered specimens from Neuquén province belonging to the subfamily Chaetocanthinae. This Argentinean species, which is undescribed, is most similar to species of the African genus *Synochodaeus* Kolbe, but there are numerous generic-level morphological differences between the Argentinean and African taxa. For that reason, a new genus and species for the Argentinean species is described below.

Larvae are unknown for the Ochodaeinae. Little is known about adults except that many species in xeric habitats are nocturnal and are attracted to light (Carlson 2001). Some rarely-seen species are diurnal and may be netted in flight or collected in pitfall traps. Flight intercept traps are the most successful technique for collecting diurnal species in forested habitats.

## Materials and methods

### Taxonomic material

Specimens examined for this study are deposited in the following institutions and private collections.

AMNH American Museum of Natural History, New York, NY, USA.

BMNH The Natural History Museum, London, UK.

CMNC Canadian Museum of Nature, Ottawa, Canada.

CMNH Carnegie Museum of Natural History, Pittsburgh, PA, USA.

CNCI Canadian National Collection of Insects, Ottawa, Canada.

DEBU University of Guelph Insect Collection, Guelph, Canada.

FMNH Field Museum of Natural History, Chicago, IL, USA.

FSCA Florida State Collection of Arthropods, Gainesville, FL, USA.

IAZA Instituto Argentino de Investigaciones de Zonas Áridas, Mendoza, Argentina.

IFML Fundación e Instituto Miguel Lillo, Tucumán, Argentina.

ISNB Institute Royal des Sciences Naturelles de Belgique, Brussels,Belgium.

LEMQ Lyman Entomological Museum, McGill University, Quebec, Canada.

MACN Museo Argentino de Ciencias Naturales, Buenos Aires, Argentina.

MJPC M.J. Paulsen Collection, Lincoln, NE, USA.

MLP Museo de la Plata, La Plata, Argentina.

UCCC Museo de Zoología de la Universidad de Concepción, Concepción, Chile.

UNSM University of Nebraska State Museum, Lincoln, NE, USA.

USNM United States National Museum (Smithsonian) Collection,Washington, DC, USA.

SARC South African National Collection, Pretoria, South Africa.

TMNH Transvaal Museum of Natural History, Pretoria, South Africa.

ZMHB Museum für Naturkunde (Humboldt Universität), Berlin, Germany.

### Morphological characters

Important characters for distinguishing species in the genus *Parochodaeus* are listed in Paulsen (2011) andinclude the armature of the head, teeth of the legs and metatrochanter, dorsal vestiture and punctation, stridulatory peg, size of the first metatarsomere, and form of the mentum. In species of *Parochodaeus* from Argentina the head armature, when present, consists of either horns, tubercles, or elevated carinae. The leg armature may consist of an acute tooth at the apex of the metafemora or metatibia, or the leg may be unarmed with only a broad, rounded lobe. In one species, the metatrochanter is also toothed. In one group of *Parochodaeus* species (‘*Parochodaeus pectoralis*-complex’ *sensu* Carlson 1975) the first tarsomere of the metatarsus is greatly enlarged and often curved. The dorsal vestiture can vary from short bristles to a longer, “shaggy” appearance, but all Argentine species display only short to moderate length setae. Each setose puncture may have an anterior tubercle, and the surface between punctures can be tuberculate or smooth, with the tubercles presenting a tiled appearance. The presence or absence of a stridulatory peg on the abdomen is often diagnostic, and to some extent the shape of the peg can be also when present, but not with the Argentine species. The width of the clypeus is more or less uniform, but the length is often species- and sex-specific. Therefore the form of the clypeus can be strongly transverse, which is described as being short (length about 1/4 or 1/5 width), or long (length about 1/2 width). The form of the mentum is important for identification and may be strongly protuberant vertically, distinctly concave, tumid, or flat. The male genitalia are weakly sclerotized and nondescript, with informative characters restricted to the sclerotized teeth and bristles of the internal sac. Unfortunately, the internal sacs are difficult to dissect if the specimens were collected directly into alcohol or were not recently collected. Because other, more tractable characters are present to distinguish species the internal sacs were not dissected for this work. Males and females do not differ appreciably in size and are not described separately, but sexually dimorphic characters, when present, are noted in the descriptions. Color is not useful in identification of the New World Ochodaeidae because most species vary from light testaceous to dark castaneous in color.

Characters for distinguishing chaetocanthines (as defined in Scholtz et al. 1988) include: 1) metatibial spurs crenulate or pectinate; 2) metatibia compressed, not cylindrical; 3) eyes not bulging; 4) galea of the maxilla rounded. These characters are problematic with respect to the new taxon, which, although clearly allied with *Synochodaeus*, does not conform to half of these criteria. In particular, the metatibial spurs of the new genus are not crenulate or pectinate in the specimens examined. Because the specimens are worn, it may be possible that the crenulations are merely eroded and were originally present, but the crenulations of the mesotibial spurs are present and have not been abraded. Also, the eyes are much larger in the new genus and are as protuberant as in many ochodaeines. The compressed metatibia is not a synapomorphy for chaetocanthines because numerous species of Ochodaeinae also display this character. The rounded galea is present, perhaps representing the strongest remaining synapomorphy for Chaetocanthinae. The new genus is placed into the tribe Synochodaeini Scholtz based on its similarity to *Synochodaeus* species in the following characters: rounded labial palps, elongate form, tridentate protibiae, anterior clypeal margin straight, antenna with ten antennomeres, first antennomere of club not strongly hemispherical, metatrochanter not acutely produced beyond posterior margin of metafemur, and sexual dimorphism lacking. However, it differs from that genus in having a much longer clypeus that is not distinctly separated from the frons by a sulcate depression and not reflexed anteriorly. In addition, the larger metatibial spur is not crenulate, the scutellum is pointed (not rounded) apically, the labrum densely punctate/setose (not mostly glabrous), and the penultimate labial palpomeres are irregularly shaped, not cylindrical.

## Taxonomic treatment

### Key to the genera of Ochodaeidae of Argentina

**Table:** 

1	Propygidium with two tubercles that interlock with acute elytral apices	*Parochodaeus* Nikolajev
1'	Propygidium unarmed; elytral apices rounded	*Gauchodaeus* Paulsen, gen. n.

### Parochodaeus

Nikolajev, 1995: 77

http://species-id.net/wiki/Parochodaeus

#### Type Species.

*Parochodaeus pectoralis* (LeConte, 1868), by original designation.

#### Diagnosis.

This genus contains species with an elytral locking mechanism consisting of a bituberculate margin on the propygidium and dentate elytral apices.

#### Description.

Ochodaeidae: Ochodaeinae. Form convex, ovate. ***Length:*** 2.6–9.1 mm. ***Width:*** 1.5–4.9 mm. ***Surface/Color:*** Dorsal surface of head, pronotum, and elytra setose; setae varying from short bristles to longer setae; setae erect or subdepressed. Color testaceous to dark castaneous, variable within species. ***Head:*** Clypeus varying from semicircular to subtrapezoidal, often sexually dimorphic in length (longer in females). Frontoclypeal suture indistinct but generally discernible. Frons simple or variably armed with tubercles, horns, or carinae. Mentum variable between species. Mandibles moderately large, externally rounded to angulate, visible beyond labrum in dorsal view. Labrum strongly projecting between mandibles, setose. Antenna with 10 antennomeres; 3- antennomere club nearly round, pubescent. ***Pronotum:*** Surface densely punctate, punctures fine to moderate, each with or without a distinct setigerous tubercle anteriorly; setae moderately long. ***Elytra:*** Striae (except sutural) not impressed, uniserially punctate; punctures moderate to large, moderately deep, round, lacking setae. Intervals tuberculate; tubercles fine to moderate, forming irregular rows, setigerous; setae variable in length. ***Legs*:** Protibia with 3 external teeth increasing in size distally; occasionally with apical internal tooth or “thumb” produced beyond tarsal insertion. Metafemur usually simple with slightly produced rounded lobe, occasionally armed with 1 large tooth or several small, irregular teeth on dorsal or ventral posterior margin before apex. Metatibia form variable from slender and subcylindrical to broad and flattened. ***Abdomen*:** Stridulatory peg, if present, near anterolateral angle of propygidium (necessary to unlock elytron and depress abdomen to view). Propygidial margin with 2 tubercles on posterior margin.

#### Composition.

The genus *Parochodaeus* currentlyconsists of 17 species known from the central Great Plains of the United States south to Río Negro Province in Argentina (Paulsen 2007; Paulsen 2011). An additional seven species from Argentina are described in this paper, bringing the total number of species in the genus to 24.

#### Remarks.

 The presence or absence of a stridulatory peg on the abdomen and the form of the mentum are extremely useful in determining species. The head armature of frontal horns, tubercles, and carinae are allometric, and, as such, extremely small specimens will still have the appropriate structure, albeit extremely reduced. In such cases, for example with exceptionally small *Parochodaeus campsognathus* (Arrow), the other characters given should be used to confirm identification.

#### Key to the Parochodaeus species of Argentina

**Table:** 

1	Vertex of head with 2 prominent tubercles in both sexes (Fig. 1)	*Parochodaeus pudu* Paulsen & Ocampo, sp. n.
1'	Vertex of head with 1 horn (Fig. 2) or tubercle, elevated carina (Fig. 3), or unarmed (anterior margin of head may have 2 clypeal tubercles)	2
2	Frontoclypeal area with distinct central horn, tubercle, or elevated carina in both sexes	3
2'	Frontoclypeal area unarmed in both sexes (*Parochodaeus jujuyus* with 2 indistinct tumosities on vertex, but these never appearing as discrete tubercles)	7
3	Frontoclypeal armature in the form of an elevated, often anteriorly pointing angular carina (Fig. 3).	*Parochodaeus campsognathus* (Arrow)
3'	Frontoclypeal armature consisting of a single horn or horn-like tubercle	4
4	Apex of metafemur toothed posteriorly (Fig. 4). Pronotal disc distinctly punctate, surface between punctures smooth	*Parochodaeus dentipes* Paulsen & Ocampo, sp. n.
4'	Apex of metafemur not toothed. Pronotal punctures obscured by roughened surface	5
5	Metatibia slender, less than 1/5 as wide as long (e.g., Fig. 6). Mentum with distinct median longitudinal furrow over entire length.	*Parochodaeus proceripes* Paulsen & Ocampo, sp. n.
5'	Metatibia broad, about 1/3 as wide as long (e.g., Fig. 4). Mentum mostly flat, at most only slightly furrowed anteriorly.	6
6	Frontoclypeal horn or tubercle with apex simple. Mentum entirely flat. Stridulatory peg present.	*Parochodaeus phoxus* Paulsen & Ocampo, sp. n.
6'	Frontoclypeal horn or tubercle with U-shaped carina at apex. Mentum sulcate anteriorly. Stridulatory peg absent	*Parochodaeus perplexus* Paulsen & Ocampo, sp. n.
7	First metatarsomere greatly expanded (Fig. 5)	*Parochodaeus jujuyus* Paulsen & Ocampo, sp. n.
7'	First metatarsomere not greatly expanded (Fig. 6)	8
8	Lateral margin of pronotum evenly rounded. Metatrochanter and apex of metafemur lacking acute teeth. Metatibia straight	*Parochodaeus cornutus* (Ohaus)
8'	Lateral margin of pronotum strongly angulate. Metatrochanter toothed, apex of metafemur with two teeth (Fig. 7). Metatibia curved	*Parochodaeus stupendus* Paulsen & Ocampo, sp. n.

### Parochodaeus campsognathus

(Arrow, 1904)

http://species-id.net/wiki/Parochodaeus_campsognathus

Figs 3 8 Map 1

Ochodaeus campsognathus Arrow 1904: 744, original combination.

#### Type material. 

Lectotype male (BMNH), pinned. Lectotype here designated to fix the concept to a single specimen from the original series. Original labels: a) “Argentina / Chaco / Wagner. / 1903-180.”; b) ♀ (sic); c) “*Ochodaeus / campsognathus*, / Type / Arrow”; d) red-bordered circular “TYPE”; e) blue-bordered circular “SYN- / TYPE”; f) “[O. campsognathus / Arrow BM / Syntype 1]” / DET. / D. C. CARLSON 19__”; g) “*Parochodaeus / campsognathus /* (Arrow, 1904) / det. M.J. Paulsen 2009”. Two paralectotypes (male and female) labeled with *a, e, g* as above specimen, and *f* except syntype numbers 2 and 3.

#### Type locality.

 Argentina: Chaco.

#### Diagnosis.

 The species is easily distinguished by its V-shaped frontal carina (Figs 3, 8), but in the smallest specimens the carinae can be almost obsolete. If necessary, the large, rectangular mentum with a complete, deep longitudinal furrow will confirm identification.

#### Description.

***Length:***4.6–9.1 mm. ***Width:***2.5–4.9mm. ***Head:*** Surface roughened as pronotum, sparsely punctate. Frons with broad V-shaped carina medially (Fig. 3), pointing anteriorly. Clypeus trapezoidal, short in males (length equal to 1/4 width), longer in females (length equal to approximately 1/3 width); anterior margin thickened, elevated but indistinctly indicated. Labrum deeply emarginate. Mandibles angulate externally, larger males with external angulation strongly produced upward. Mentum large, rectangular, with distinct longitudinal furrow for entire length. ***Pronotum:*** Form convex to strongly convex in larger individuals. Surface with densely tiled tubercles, tubercles moderate; surface between tubercles punctate; punctures fine. ***Elytra:*** Setae of interstrial tubercles moderately long, erect. ***Legs:*** Protibia with apical spur weakly curved; internal apical tooth lacking. Metatrochanter simple. Metafemur with posterior margin simple. Metatibia straight, narrow (>4× longer than wide) expanding gradually to apex. Metatarsomere 1 not greatly enlarged. ***Abdomen:***Stridulatory peg present.

**Figures 1–3.**
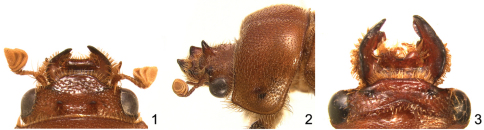
Characters of the head of *Parochodaeus* species **1** Head of *Parochodaeus pudu* with 2 frontal tubercles, dorsal view **2** Head and pronotum of *Parochodaeus procelipes*, lateral viewshowing clypeal horn and pronotum that is strongly declivous anteriorly **3** Head of *Parochodaeus campsognathus* with large, angulate carina on vertex of head, dorsal view

#### Distribution

**Map 1.**
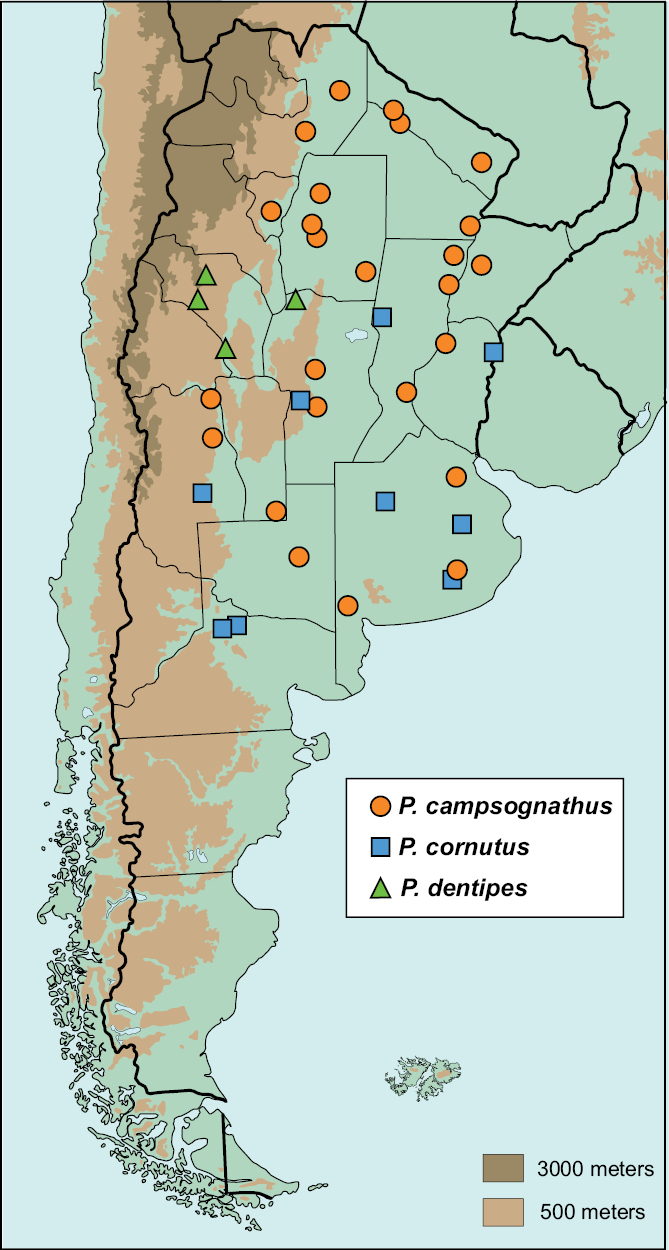
Argentinean distribution of *Parochodaeus campsognathus* (circles), *Parochodaeus cornutus* (squares), and *Parochodaeus dentipes* (triangles)

(Map 1). 153 specimens examined.

**ARGENTINA:** BUENOS AIRES: Lobos Estancia El Ombú (1),Puán (1), Tandil (1); CHACO: Resistencia (100 km NW) (1); CÓRDOBA: Anisacate (2), Río Cuarto (45 km N) (4), no locality (17); CORRIENTES: San Roque (1); ENTRE RÍOS: Santa Elena (2); FORMOSA: Ingeniero Juárez (1), Gran Guardia (1), Pilcomayo (1), No data (2); LA PAMPA: General Acha (1); MENDOZA: El Retamo (1), Moliches (7), Reserva de la Biósfera Ñacuñán (2), no locality (2); SAN LUIS: Arizona (7); SALTA: Joaquín V. González (1), Rivadavia (1); SANTA FÉ: Estancia la Noria (17), Lanteri (3), Villa Ana (7); SANTIAGO DEL ESTERO: El Pinto (1), Fernández (4), La Banda (1), Ojo de Agua (1), Río Salado (28); TUCUMÁN: Encrucijada (3), no locality (1). No data (4).

**BOLIVIA:** SANTA CRUZ: Cordillera Parapetí (1).

**BRAZIL:** MATO GROSSO: Cuiabá (2); RIO GRANDE DO SUL: Rio Grande do Chapada (1). No data (2).

**PARAGUAY:** BOQUERÓN: 145 km from Puerto Casado (3); Guairá: Independencia (1).

**Temporal distribution**.January (16), February (16), March (10), April (3), October (1), November (6), December (25). No data (70).

#### Remarks.

Based on the collections examined, this is the most commonly collected species of ochodaeid in South America, and it is also the largest.

The original description of Arrow (1904) mentioned six syntypes, although there are ten specimens with the appropriate Wagner labels in the Natural History Museum in London. There are two pairs, (2♂, 2♀), treated as syntypes by Carlson, who labeled them numbers 1–4. One male with a BMNH type label (Syntype #1 *sensu* Carlson) had the genitalia dissected by Carlson, and this specimen is chosen as the lectotype. The fourth specimen (Carlson’s Syntype #4) labeled “Argentina / Rio las Garzas / E. Wagner. / 1907-384” is a female accessioned three years after Arrow’s publication. Because this date is after the publication date, it is not clear that Arrow studied the specimen and its syntype status is questionable.

### Parochodaeus cornutus

(Ohaus, 1910)

http://species-id.net/wiki/Parochodaeus_cornutus

Fig. 9 Map 1

Ochodaeus cornutus Ohaus 1910: 174, original combination.

#### Type material. 

Lectotype male (MACN), pinned. Lectotype here designated to fix the concept to a single specimen from the original series. Original labels: a) black-bordered “Rep. Argentina / Prov. Buenos Aires / 190_ / C. Bruch”; b) ♂; c) “Cotypus” on pale green paper; d) red-bordered “*Ochodaeus* / *cornutus* / Ohaus” handwritten by Bruch; e) “*Ochodaeus* / *cornutus* Ohs. / ♂ / Det. F. Ohaus 1909”, with “MACN-En 1019” on reverse; f) red paper “*Ochodaeus / cornutus* ♂ / Ohaus, 1910 / LECTOTYPE / Det. Paulsen & Ocampo”. Paralectotype (MACN), labels a-e as lectotype except with ♀ when appropriate, and “MACN-En 1020”; f) yellow paper “*Ochodaeus / cornutus ♀ /* Ohaus, 1910 / PARALECTOTYPE / Det. Paulsen & Ocampo”.

#### Type locality.

 Argentina: Buenos Aires and Santa Fé.

#### Diagnosis.

 Males of *Parochodaeus cornutus* (Fig. 9) are quickly distinguishable due to the two tubercles on the anterior clypeal margin. Females lack tubercles, but the simply tumid mentum combined with unarmed metatibiae will confirm identification when not associated with males.

#### Description.

***Length:***5.7–8.0 mm. ***Width:***2.8–4.0mm. ***Head:*** Surface variably roughened, tuberculate or not, sparsely punctate. Frons with 2 low tumosities. Clypeus semicircular, long (length equal to 1/2 width), anterior margin of males with an erect, horn-like tubercle on each end, margin between tubercles often indistinct; females lacking tubercles. Labrum emarginate. Mandibles rounded externally. Mentum tumid, lacking longitudinal furrow. ***Pronotum:*** Form convex, strongly so in males (declivous anteriorly). Surface tuberculate, tubercles moderate to large; surface between tubercles punctate; punctures moderate. ***Elytra:*** Setae of interstrial tubercles short, erect. ***Legs:*** Protibia with apical spur nearly straight; internal apical tooth lacking. Metatrochanter simple. Metafemur with posterior margin simple. Metatibia straight, narrow (>4× longer than wide) expanding gradually to apex. Metatarsomere 1 not greatly enlarged. ***Abdomen:***Stridulatory peg absent.

#### Distribution

(Map 1). 30 specimens examined.

**ARGENTINA:** BUENOS AIRES: El Jabalí (1), Rosas(3), Tandil/Lonacepín (1), No data (2); CÓRDOBA:Alpa Corral Estancia Eloísa (1); ENTRE RÍOS: Concordia (6); MENDOZA: General Alvear (1); NEUQUÉN: No locality (2); SANTA FÉ: Arrufo (2); RÍO NEGRO: Catriel (4), Coronel Gómez (1).

**BRAZIL:** RIO GRANDE DO SUL: Pelotas (1).

**PARAGUAY:** CORDILLERA: San Bernardino (3); VILLARICA: Guairá (2).

**Temporal distribution**.January (10), February (2), June (2), July (4), October (1), November (2). No data (5).

#### Remarks.

 Ohaus (1910) examined adults of both sexes from the Bruch and Richter collections, from Buenos Aires and Santa Fé respectively. Two Bruch specimens in the MACN collection are labeled as syntypes, and we designate the male as the lectotype. No specimens from ZMHB, the depository of many Ohaus types, are potential syntypes. Thus, the Richter syntypes have not been located.

### Parochodaeus dentipes

Paulsen & Ocampo p. n.

urn:lsid:zoobank.org:act:61A3371B-6765-48B7-916B-9DDAEB26895E

http://species-id.net/wiki/Parochodaeus_dentipes

Figs 4 10 Map 1

#### Type material.

 Holotype male (MLP), pinned. Original labels: a) “Piedra Pintada / La Rioja 23-II-39 / Birabén-Scott leg.” b) red paper “*Parochodaeus* / *dentipes* / Paulsen & Ocampo / HOLOTYPE”. One paratype (MLP) labeled as holotype. Three paratypes (LEMQ) labeled: a) “ARGENTINA, La Rioja / Guandacol, 1000m / 1-3.XII.1983, L.E. Peña / at light”; b) black-bordered “Lyman Entomological / Museum / Ste-Anne-Bellevue / Canada”. One paratype (CMNC) labeled: a) “ARGT / La Rioja / Mascasín / I- 1959”. One paratype (CMNC) labeled “Argentina / Cordoba / Do. Cruz del Eje / Guanaco Muerto / Coll. Martínez” and “Ene. 977”. All paratypes with “*Parochodaeus* / *dentipes* / Paulsen & Ocampo / PARATYPE” on yellow paper.

#### Type locality.

 Argentina: La Rioja: Piedra Pintada.

#### Diagnosis.

**Figures 4–7.**
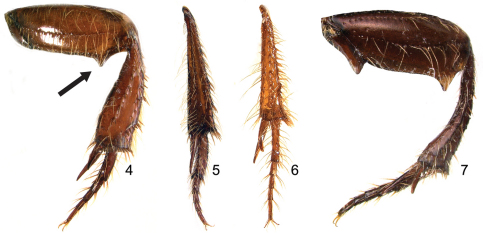
Characters of the left hindleg of *Parochodaeus* species, ventral view **4** *Parochodaeus dentipes* with arrow indicating acute tooth near apex of metafemur; metatibia broad **5** *Parochodaeus jujuyus*, showing greatly enlarged first metatarsomere **6** *Parochodaeus procelipes*, narrow metatibia **7** *Parochodaeus stupendus*, with large metatrochanteral tooth, large ventral tooth at the metafemoral apex (second, dorsal tooth obscured), and strongly curved metatibia.

 This is one of only two species in Argentina with a large tooth at the apex of the metafemur (Fig. 4), and the tooth is present in both sexes. The species can be distinguished by the presence of that tooth and a frontal horn on the head (Fig. 10).

#### Description, holotype male. 

***Length:***7.7 mm. ***Width:***4.0mm. ***Head:*** Surface tuberculate, punctate; punctures moderate. Frons with central tubercle. Clypeus broadly rounded, narrow, short (length equal to 1/5 width); anterior margin thickened, produced anteriorly in both sexes. Labrum deeply emarginate. Mandibles weakly angulate externally. Mentum tumid, notched anteriorly. ***Pronotum:*** Form convex. Surface shiny, weakly tuberculate anteriorly, tubercles obsolete on disc; surface between tubercles punctate; punctures moderate. ***Elytra:*** Setae of interstrial tubercles moderately long, decumbent. ***Legs:*** Protibia with apical spur weakly curved; internal apical tooth present; tooth short, acute. Metatrochanter simple. Metafemur with tooth on posterior margin before apex. Metatibia curved (in ventral view external margin rounded, internal margin sinuate), broad (at apex approximately as wide as mesofemur), expanding abruptly in basal third. Metatarsomere 1 not greatly enlarged. ***Abdomen:***Stridulatory peg absent.

**Description, paratypes (n=6).** ***Length:***6.5–7.6 mm. ***Width:***3.3–3.9 mm. Differs from holotype in the following external characters: ***Head:*** Females with clypeus longer, length equal to 1/4 width.

**Figures 8–10.**
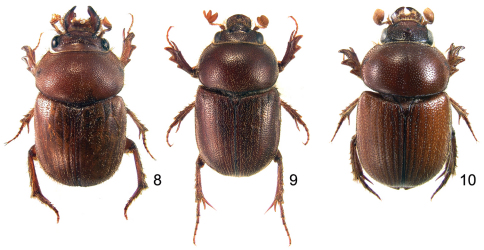
Dorsal habitus of *Parochodaeus* species, males **8** *Parochodaeus campsognathus* **9** *Parochodaeus cornutus* **10** *Parochodaeus dentipes*, sp. n.

#### Etymology.

The specific epithet ‘dentipes’ is from Latin *dentis* “tooth” and *pes* “foot, leg”, for the toothed metafemur, and is used a masculine adjective in the nominative singular.

#### Distribution

(Map 1). 7 specimens examined.

**ARGENTINA:** CÓRDOBA: Cruz del Eje (1); LA RIOJA: Guandacol (2), Mascasín (1), Piedra Pintada (2).

#### Temporal distribution.

January (2), February (2), December (2).

#### Remarks.

Only seven specimens of this species are known. The specimens from Guandacol were collected at light.

### Parochodaeus jujuyus

Paulsen & Ocampo sp. n.

urn:lsid:zoobank.org:act:1156FB0B-89D1-4D3E-9E0F-8FFD9D10D659

http://species-id.net/wiki/Parochodaeus_jujuyus

Figs 5 11 Map 2

#### Type material.

 Holotype male (CMNC), pinned. Original labels: a) “ARG: Jujuy Prov. / Calilegua Nat. Park / 18-28. XII. 87, S&J Peck / El Cortaderal, km 6, 800m / forest malaise-FIT”; b) on red paper “*Parochodaeus* / *jujuyus* / ♂ /Paulsen & Ocampo / HOLOTYPE”. Allotype female (CMNC) labeled: a) “ARGENTINA / SALTA / D° Anta / El Rey / Bordón- leg. / Coll. Martínez / Feb. 984”; b) “*Parochodaeus* / *jujuyus* / ♀ /Paulsen & Ocampo / ALLOTYPE” on red label. Two paratype males (DEBU) labeled: a) ARGENTINA: Salta / 30km E Campo Quijano / FIT; 18-28.ii.1992 / S. A. Marshall”; b) “*Parochodaeus* / *jujuyus* /Paulsen & Ocampo / ♂ /PARATYPE” on yellow label.

#### Type locality.

 Argentina: Jujuy: Calilegua National Park.

#### Diagnosis.

**Figures 11–13.**
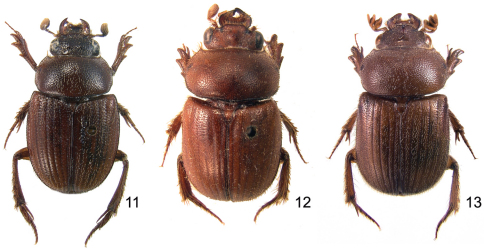
Dorsal habitus of *Parochodaeus* species, males **11** *Parochodaeus jujuyus*, sp. n.**12** *Parochodaeus perplexus*, sp. n.**13** *Parochodaeus phoxus*, sp. n.

*Parochodaeus jujuyus* (Fig. 11) is the only Argentine species of the *Parochodaeus pectoralis* group. Members of this species group have greatly enlarged first metatarsomeres (Fig. 5) and often have the mentum produced ventrally.

#### Description, holotype male. 

***Length:***7.0 mm. ***Width:***3.5mm. ***Head:*** Surface weakly tuberculate, shiny, punctate; punctures moderate, setose. Frons unarmed but with 2 indistinct tumosities. Clypeus broadly rounded, narrow, short (length equal to 1/4 width), margin thickened, produced anteriorly, as long as remainder of clypeus. Labrum emarginate. Mandibles rounded externally. Mentum deeply furrowed, produced downward laterally. ***Pronotum:*** Form convex. Surface with weakly tuberculate, setose punctures mixed with glabrous punctures; punctures moderate in size. ***Elytra:*** Setae of interstrial tubercles moderately long, erect, somewhat abraded. ***Legs:*** Protibia with apical spur weakly curved; internal apical tooth absent. Metatrochanter simple. Metafemur with posterior margin simple. Metatibia slender, (~5× longer than wide) gradually widening to apex. Metatarsomere 1 subrectangular (not cylindrical), greatly enlarged (longer than greatest width of metatibiae), weakly curved. ***Abdomen:***Stridulatory peg present.

**Description, allotype female.** ***Length:***6.0 mm. ***Width:***3.0 mm. Differs from male holotype in the following external characters: ***Head:***Clypeus longer (length equal to 1/2 width); anterior margin not produced anteriorly. ***Elytra:***Setae more abraded.

**Description, paratypes (n=2).** ***Length:***5.8–6.6 mm. ***Width:***3.1–3.3 mm. Not differing significantly from the holotype except with dorsal vestiture less abraded.

#### Etymology.

The specific epithet ‘jujuyus’ is an unconventional but euphonious Latinized form of the province name Jujuy, gender masculine.

#### Distribution

**Map 2.**
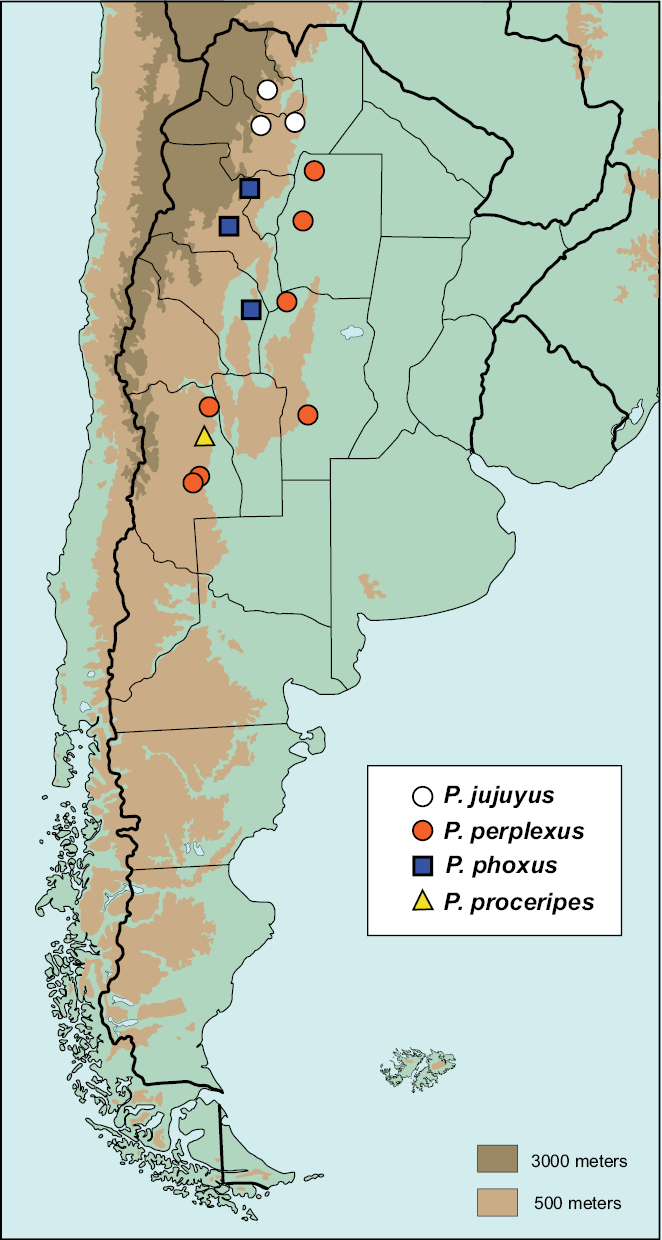
Argentinean distribution of *Parochodaeus jujuyus* (white circles), *Parochodaeus perplexus* (orange circles), *Parochodaeus cornutus* (blue squares), and *Parochodaeus dentipes* (yellow triangle).

(Map 2). 4 specimens examined.

**ARGENTINA:** JUJUY: Parque Nacional Calilegua El Cortaderal (1); SALTA: Campo Quijano (30 Km E) (2), Parque Nacional El Rey (1).

#### Temporal distribution.

February (3), December (1).

#### Remarks.

This species is known from only four specimens and is from the far north of Argentina. The habitat in the region is montane forest. Label data indicates that the paratypes were collected in flight-intercept traps, which is the most common method of collection for forest ochodaeids.

### Parochodaeus perplexus

Paulsen & Ocampo sp. n.

urn:lsid:zoobank.org:act:0A6E2DAC-595E-4BA2-96E2-70DA1F59E12D

http://species-id.net/wiki/Parochodaeus_perplexus

Fig. 12 Map 2

#### Type material.

 Holotype male (MLP), pinned. Original labels: a) “Sgo del ESTERO / RIO SALADO / Wagner Col.” b) “*Parochodaeus* / *perplexus* /Paulsen & Ocampo / HOLOTYPE” on red paper. One paratype (CNCI) labeled: a) “ARGT. Stgo. / del Estero”; b) “Fernández / II 1957”; c) “*Parochodaeus* / *campsognathus* / (Arrow) / Vaz-de-Mello det 2003”. One paratype at (ZMHB) labeled: “Bolivien / Chaco de Bolivia / K. Pflanz S.G.”. One paratype (ZMHB) labeled: a) “Rio Cuarto / Argentina / Breuer”; b) “80.” One paratype (MJPC) labeled: a) “ARGT. Santi. del / Estero, Fernández, / II 1957”; b) “*Parochodaeus* / *campsognathus* / (Arrow) / Vaz-de-Mello det 2003”. One paratype (IAZA) labeled: “Mendoza, Lavalle / 20 km W de Arroyito / 12-XII-1979 / Col. A. Roig / SERGIO ROIG”. One paratype (IAZA) labeled: “San Rafael / Los Toldos, 51 km / SW de Soitué / 31-I-1979 / Sergio Roig.” One paratype (CMNC) labeled: “ARGENTINA / CÓRDOBA / D° Cruz del Eje / Guanaco Muerto / Coll. Martínez / Feb. 980”. Two paratypes (IAZA) labeled: “San Rafael / Huarpes / 2-II-79 / Sergio Roig”. All paratypes labeled: “*Parochodaeus* / *perplexus* /Paulsen & Ocampo / PARATYPE” on yellow paper.

#### Type locality.

 Argentina: Santiago del Estero: Río Salado.

#### Diagnosis.

 The species is similar to *Parochodaeus dentipes* in that both species have a frontal ‘horn’ and broad metatibiae. *Parochodaeus perplexus* (Fig. 12) can be separated by its lack of a large tooth at the apex of the metafemur and by the flatter mentum.

#### Description, holotype male.

***Length:***6.9 mm. ***Width:***3.8mm. ***Head:*** Surface tuberculate, punctate; punctures small. Frons with central horn-like tubercle; tubercle with apex U-shaped. Clypeus subtrapezoidal, narrow, short (length equal to 1/4 width); anterior margin not thickened, truncate. Labrum broadly emarginate. Mandibles angulate externally. Mentum flat, weakly concave anteriorly surrounding longitudinal furrow in anterior half. ***Pronotum:*** Form convex. Surface with densely tiled tubercles; tubercles small, setose; surface between tubercles punctate; punctures fine. ***Elytra:*** Setae of interstrial tubercles short, erect. ***Legs:*** Protibia with apical spur weakly curved; internal apical tooth absent. Metatrochanter simple. Metafemur with posterior margin simple. Metatibia weakly curved (in ventral view external margin rounded, internal margin sinuate), broad (at apex approximately as wide as mesofemur), expanding abruptly in basal third. Metatarsomere 1 not greatly enlarged. ***Abdomen:***Stridulatory peg absent.

**Description, paratypes.** ***Length:***5.0–7.9 mm. ***Width:***2.9–4.2 mm. Differing from the holotype in the following external characters: ***Head:*** Female paratypes with clypeus longer, length equal to 1/3 width.

#### Etymology.

The specific epithet ‘*perplexus*’ is Latin meaning “muddled, interwoven”, because this species displays a mix of characters present in other species. The name also aptly represents how perplexed we were to find yet another new species from central Argentina. It is used a masculine adjective in the nominative singular.

#### Distribution

(Map 2). 10 specimens examined.

**ARGENTINA:** CÓRDOBA: Guanaco Muerto (1), Río Cuarto (1); MENDOZA: Lavalle (20 Km W Arroyito) (1), Los Toldos, 51 Km SW Soitué (1), San Rafael (2); SANTIAGO DEL ESTERO: Fernández (2), Río Salado (1).

**BOLIVIA: “**Chaco de Bolívia” (1).

#### Temporal distribution.

January (1), February (5), December (1), No data (3).

#### Remarks.

Ten specimens of this species are known. None of the labels have any information on how they were collected, but given the open habitat of the collecting localities they were probably taken at light.

### Parochodaeus phoxus

Paulsen & Ocampo sp. n.

urn:lsid:zoobank.org:act:13AB2413-5166-41E5-9DC4-51F3F948FC91

http://species-id.net/wiki/Parochodaeus_phoxus

Fig. 13 Map 2

#### Type material.

 Holotype male (FSCA), pinned. Original labels: a) “ARGENTINA: LA RIOJA / Castro Barros; Santa Vera / Cruz; b) -28.67 -66.96; 1600m / 15-II-2005; L. Stange”; b) red paper “*Parochodaeus* / *phoxus* / ♂ / Paulsen & Ocampo / HOLOTYPE”. Three paratypes (FSCA, MJPC) labeled as holotype. Four paratypes (UNSM, IAZA) labeled: “ARGENTINA: Catamarca: / Londres. 15 km / SW Belen / 5 Dec. 1973 - 24 Jan. 1974 / Frank A. Enders”. One paratype (USNM) labeled: a) “ARGENTINA: Tucumán / Amaichá del Valle, 1995 m. / S 26º 35’ 22” W 65º 55’ 13” / XII-7-2003 F.C. Ocampo”; b) black-bordered “UNSM SCARAB DNA / VOUCHER SPECIMEN / [FO 63 4/04]”. All paratypes labeled: “*Parochodaeus* / *phoxus* /Paulsen & Ocampo / PARATYPE” on yellow paper.

#### Type locality.

 Argentina: La Rioja: Castro Barros, Santa Vera Cruz.

#### Diagnosis.

*Parochodaeus phoxus* (Fig. 13) is similar to *Parochodaeus proceripes* in that it also has a large frontal horn (Fig. 2). In *Parochodaeus phoxus*, the flatter mentum lacking a longitudinal furrow and broad metatibiae will immediately separate it from *Parochodaeus proceripes*, which has a distinctly furrowed mentum and slender metatibiae.

#### Description, holotype male.

***Length:***7.6 mm. ***Width:***4.0mm. ***Head:*** Surface tuberculate, punctate; punctures small. Frons with horn. Clypeus subtrapezoidal, narrow, short (length equal to 1/4 width); anterior margin not thickened, truncate. Labrum broadly emarginate. Mandibles obtusely angulate at basal third. Mentum almost flat, weakly concave, declivous anteriorly, lacking longitudinal furrow. ***Pronotum:*** Form strongly convex, declivous anteriorly. Surface densely tiled with tubercles; tubercles small, setose; surface between tubercles punctate; punctures fine. ***Elytra:*** Setae of interstrial tubercles moderately long, erect. ***Legs:*** Protibia with apical spur weakly curved; internal apical tooth absent. Metatrochanter simple. Metafemur with posterior margin simple. Metatibia straight, moderately broad (at apex not as wide as mesofemur), gradually expanding to apex. Metatarsomere 1 not greatly enlarged. ***Abdomen:***Stridulatory peg present.

**Description, paratypes** **(n=8). *Length:***6.4–8.0 mm. ***Width:***3.5–4.1 mm. Differing from the holotype in the following external characters: ***Head:*** Female paratypes with clypeus longer, length equal to 1/3 width.

#### Etymology.

The specific epithet ‘*phoxus*’ is Latinized form of the Greek ‘*phoxos’*, or “pointed, peaked”, referring to the frontal horn. It is used a masculine adjective in the nominative singular.

#### Distribution

(Map 2). 9 specimens examined.

**ARGENTINA:** CATAMARCA: Londres (4);LA RIOJA: Santa Vera Cruz (4); TUCUMÁN: Amaichá del Valle (1).

#### Temporal distribution.

February (4), December (5).

#### Remarks.

Only nine specimens of this species are known. Nothing is known about the life history of these beetles. The Tucumán specimen was collected at mercury vapor light.

### Parochodaeus proceripes

Paulsen & Ocampo sp. n.

urn:lsid:zoobank.org:act:36E307A5-43C6-4F0A-B54F-77959A7C8BF1

http://species-id.net/wiki/Parochodaeus_proceripes

Figs 2 6 14 Map 2

#### Type material.

 Holotype male (IAZA), pinned. Original labels: a) “RA Mza Santa Rosa / Ñacuñán tramp luz UV / 12-X-02 G. Flores”; b) red paper “*Parochodaeus* / *proceripes* / ♂ / Paulsen & Ocampo / HOLOTYPE”.

#### Type locality.

 Argentina: Mendoza: Santa Rosa.

#### Diagnosis.

**Figures 14–16.**
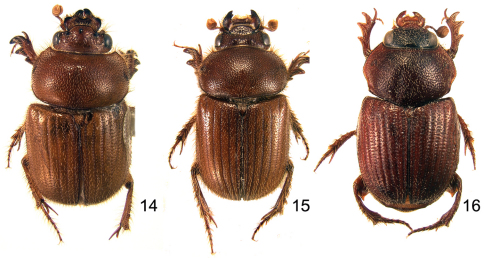
Dorsal habitus of *Parochodaeus* species, males **14** *Parochodaeus procelipes* sp. n. **15** *Parochodaeus pudu*, sp. n.**16** *Parochodaeus stupendus*, sp. n.

*Parochodaeus proceripes* (Fig. 14) is similar to *Parochodaeus phoxus* in that it also has a large frontoclypeal horn, but the horn of this species is larger, more anterior, and is almost entirely located on the clypeus. The slender metatibiae are unique among horned species in Argentina.

#### Description, holotype male.

***Length:***8.1 mm. ***Width:***4.2mm. ***Head:*** Surface shiny, weakly tuberculate, punctate; punctures small, sparse. Vertex with frontoclypeal horn (Fig. 2). Clypeus subtrapezoidal, long (length equal to 1/2 width); anterior margin not thickened, broadly rounded. Labrum broadly emarginate. Mandibles strongly angulate externally. Mentum with distinct longitudinal furrow for entire length. ***Pronotum:*** Form strongly convex, declivous anteriorly. Surface densely tiled with tubercles; tubercles moderate in size, setose; surface between tubercles punctate; punctures fine. ***Elytra:*** Setae of interstrial tubercles short, erect. ***Legs:*** Protibia with apical spur curved externally; internal apical tooth absent. Metatrochanter simple. Metafemur with posterior margin simple. Metatibia straight, slender (width approximately 1/5 length; Fig. 6), gradually expanding to apex. Metatarsomere 1 not greatly enlarged. ***Abdomen:***Stridulatory peg present.

#### Etymology.

The specific epithet ‘*proceripes*’ is derived from the Latin *procerus* “long, slender” and *pes* “foot, leg”, referring to the slender metatibiae, which are unusual in species with a frontoclypeal horn. The name is used a masculine adjective in the nominative singular.

#### Distribution

(Map 2). 1 specimen examined.

**ARGENTINA**: MENDOZA: Santa Rosa- Reserva de la Biósfera Ñacuñán (1).

#### Temporal distribution.

October (1).

#### Remarks.

Only the holotype is known, which is male. The specimen was collected at a light trap.

### Parochodaeus pudu

Paulsen & Ocampo sp. n.

urn:lsid:zoobank.org:act:C6B6C3D6-858A-47A9-A26E-CC040F5BAA80

http://species-id.net/wiki/Parochodaeus_pudu

Figs 1 15 Map 3

#### Type material.

 Holotype male (IAZA), pinned. Original labels: a) “ARGENTINA: La Pampa / General Acha. / 15-III-2008. at light / Col. D. Carpintero” ; b) red paper “*Parochodaeus* / *pudu* / ♂ / Paulsen & Ocampo / HOLOTYPE”. Allotype female (IAZA) with a) as holotype; b) “*Parochodaeus* / *pudu* / ♀ / Paulsen & Ocampo / ALLOTYPE” (red label). Twenty-one paratypes (IAZA, MJPC, UNSM) labeled a) as holotype. Four paratypes (CMNC) labeled: a) “ARGENTINA / Córdoba”; “Ao. TEGUA / 5, 9-Abril-1967”; b) Coll: L.E. Peña / y G. Barriga”; c) “H. & A. Howden / Collection / *ex*. A. Martínez coll.”. One paratype (FSCA) labeled: “ARGENTINA: LA RIOJA / Castro Barros: Santa Vera / Cruz; -28.67 -66.96; 1600m / 30-II-2004; L. Stange”. One paratype (MJPC) labeled: “ARGENTINA: LA RIOJA / Castro Barros: Santa Vera / Cruz; -28.67 -66.96; 1600m / 15-II-2005; L. Stange”. Three paratypes (CMNC) labeled: a) “ARGENTINA / Jujuy / CIUDAD / Patología / Prosen - legit / Coll. Martínez / May 948”; b) “H. & A. Howden / Collection / *ex*. A. Martínez coll.” One paratype (CMNC) labeled: a) “ARGENTINA / Jujuy / S.S. de Jujuy / Prosen – legit / Coll. Martínez”; b) “H. & A. Howden / Collection / *ex*. A. Martínez coll.” One paratype (CMNC) labeled: a) “ARGENTINA / SALTA / D° G. San Martín / Pocitos / Coll. Martínez”; b) “H. & A. Howden / Collection / *ex*. A. Martínez coll.” One paratype (IFML) labeled: a) “ARGENTINA / TUCUMÁN / 11 km. cerca / de Las Cejas”; b) “Trampa malaise”. Two paratypes (CMNC) a) labeled: “ARGENTINA / Buenos Aires / D° de Puán / Felipe Solá / Coll. Martínez / Ene. 949”; b) “H. & A. Howden / Collection / *ex*. A. Martínez coll.” One paratype (CMNC) labeled: a) “ARGENTINA / Buenos Aires / D° de Puán / Felipe Solá / Coll. Martínez / Ene. 949”; b) “*Ochodaeus* / *campsognathus* / Arr. G. J. Arrow. det.”; c) “H. & A. Howden / Collection / *ex*. A. Martínez coll.” Four paratypes (FMNH) labeled: “Argent. Fama - / balastro: 10. / 3.22. Weiser”; FIELD MUS / (F. Psota Coll.)”. Three paratypes (CMNC) labeled: a) “ARGENTINA / Prov. Córdoba / Abril-1967”; “Coll. L.E. Peña / y G. Barriga”; b) “H. & A. Howden / Collection / *ex*. A. Martínez coll.” One paratype (UCCC) labeled: a) “Argentina / Los Ángeles / Catamarca / II-46 / Bosq.”; b) “5751”. One paratype (IAZA) labeled:  “ARGENTINA: San Luis / San Gerónimo (500 m NW) / 33°45'52"S, 66°31'31"W. / 546m. 6/IV/10. / F.C. Ocampo, S. Roig”. Three paratypes (MLP) labeled: “Caspinchango / Catam. 10-III-921”. One paratype (MLP) labeled: “La Rioja / Huanchín / 1925”. All paratypes labeled: “*Parochodaeus* / *pudu* /Paulsen & Ocampo / PARATYPE” on yellow paper.

#### Type locality.

 Argentina: La Pampa: General Acha.

#### Diagnosis.

*Parochodaeus pudu* (Fig. 15) is the only species of *Parochodaeus* with two small horns on the vertex of the head (Fig. 1). Because of this, the species is immediately recognizable. In *Parochodaeus bituberculatus* (Erichson) of Peru the frontal structures are not horn-like, being instead two short, transverse carinae.

#### Description, holotype male.

***Length:***7.2 mm. ***Width:***3.8mm. ***Head:*** Surface shiny, roughened near eyes, punctate; punctures small. Frons with two horn-like tubercles on vertex. Clypeus long (length equal to 1/2 width), depressed basally, almost foveate; anterior margin semicircular, not thickened, often indistinct. Labrum subtruncate. Mandibles externally rounded. Mentum tumid, eroded anteriorly, lacking longitudinal furrow. ***Pronotum:*** Form strongly convex, not declivous anteriorly. Surface densely tiled with tubercles; tubercles small, setose; surface between tubercles punctate; punctures small. ***Elytra:*** Setae of interstrial tubercles short, erect. ***Legs:*** Protibia with apical spur curved; internal apical tooth absent. Metatrochanter simple. Metafemur with posterior margin simple. Metatibia straight, slender (width approximately 1/5 length), gradually expanding to apex. Metatarsomere 1 not greatly enlarged. ***Abdomen:***Stridulatory peg absent.

**Description, allotype female.** ***Length:***6.7 mm. ***Width:***3.4 mm. Differs from male holotype in the following external characters: ***Pronotum:***Form not as strongly convex.

**Description, paratypes** **(n=50). *Length:***5.1-–8.8 mm. ***Width:***2.5–4.5 mm. Not differing significantly from the holotype and allotype in external characters.

#### Etymology.

The frontal horns are reminiscent of those found on pudú, the small South American deer, *Pudu puda* (Molina). The name ‘*pudu*’ is used as a masculine noun in the nominative singular.

#### Distribution

**Map 3.**
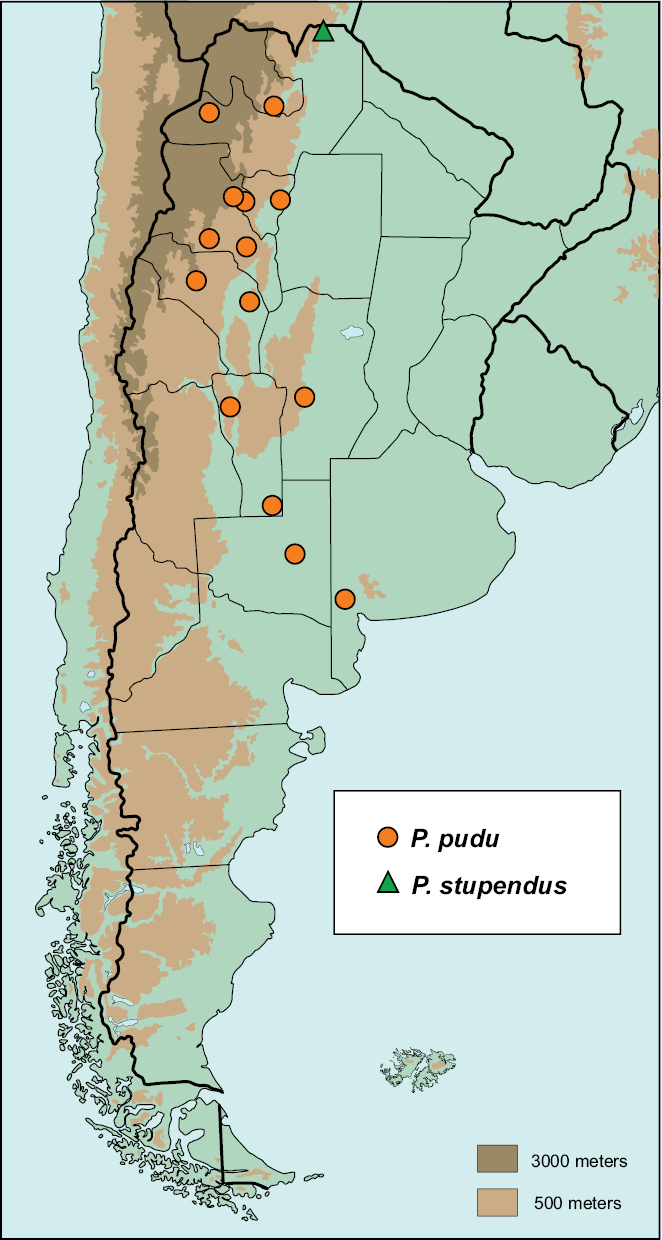
Argentinean distribution of *Parochodaeus pudu* (orange circles), and *Parochodaeus stupendus* (green triangle).

(Map 3). 52 specimens examined.

**ARGENTINA:** BUENOS AIRES: Felipe Solá (3); CATAMARCA: Caspichango (6), Famabalasto (1); Los Ángeles (1); CÓRDOBA: No locality (3); JUJUY: San Salvador de Jujuy (1), “Ciudad Patología” (3); LA PAMPA: General Acha (23); LA RIOJA: Huanchín (1), Santa Vera Cruz (2), Tegua (4); SALTA: Pocitos (1); SAN LUIS: Arizona- 18 Km S (1), San Gerónimo (1); TUCUMÁN: Las Cejas (1).

#### Temporal distribution.

January (1), February (6), March (26), April (8), May (4). No data (1).

#### Remarks.

This species is relatively well represented in collections. It is surprising that such a distinctive species remained undescribed for so long. With the two small horns on the head it is immediately recognizable and cannot be confused with any other species in the genus. The life history of the species is mostly unknown. Label data indicate that many specimens were collected at light traps, which at the very least infers a nocturnal habit for the species.

### Parochodaeus stupendus

Paulsen & Ocampo sp. n.

urn:lsid:zoobank.org:act:523C896D-1CFC-4281-824D-F2C2BAF3DD7D

http://species-id.net/wiki/Parochodaeus_stupendus

Figs 7 16 Map 3

#### Type material.

 Holotype male (CMNC), pinned. Original labels: a) “ARGENTINA / SALTA / D° San Martín / Hito 1 / Coll. Martínez / Dic 971”; b) red label “*Parochodaeus* / *stupendus* /Paulsen & Ocampo / HOLOTYPE”.

#### Type locality.

 Argentina: Salta: San Martín, Hito 1.

#### Diagnosis.

**Figure 17.**
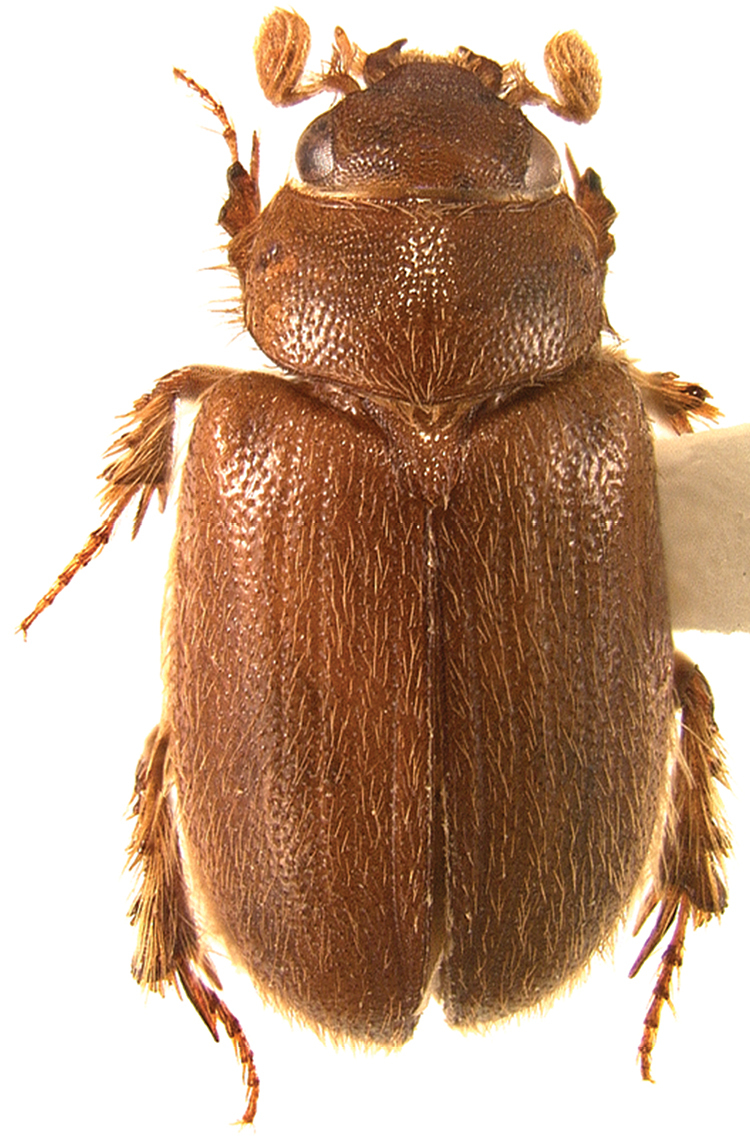
Dorsal habitus of *Gauchodaeus patagonicus* gen. n., sp. n.

*Parochodaeus stupendus* (Fig. 17) is the only species of *Parochodaeus* to exhibit a doubly-toothed posterior margin on the metafemur. The tooth on the ventral surface is fairly common in ochodaeids, but the second tooth on the dorsal surface is unique. The toothed metatrochanter and strongly curved metatibiae (Fig. 7) are not found in any other species in Argentina, or elsewhere. The strongly produced ‘thumb’ (internal apical tooth) is also species specific. The female is unknown.

#### Description, Holotype.

***Length:***7.5 mm. ***Width:***4.1 mm. ***Head:*** Surface tuberculate, tubercles moderately large, setose. Frons unarmed but indistinctly tumid medially. Clypeus evenly rounded, long (length equal to 1/2 width), margin thickened, produced anteriorly, declivous. Labrum emarginate. Mandibles rounded externally. Mentum tumid, not deeply furrowed. Antennal club strongly globose (Fig. 12). ***Pronotum:*** Form convex. Surface with moderately large, setose tubercles mixed with glabrous punctures; punctures moderate in size, sparse. ***Elytra:*** Setae of interstrial tubercles moderately long, erect, somewhat abraded. ***Legs:*** Protibia with apical spur strongly curved externally; internal apical tooth distinctly produced, subequal to basal protibial tooth in size. Metatrochanter with acute tooth. Metafemur with posterior margin acutely toothed on both dorsal and ventral surfaces. Metatibia strongly curved, slender, abruptly widening in distal third. Metatarsomere 1 not greatly enlarged. ***Abdomen:***Stridulatory peg absent.

#### Etymology.

The specific epithet ‘*stupendus*’ is Latin meaning “causing astonishment or wonder”. With several autapomorphies not seen in other species of the genus, *Parochodaeus stupendus* is arguably the most wondrous ochodaeid in the New World. The name is used a masculine adjective in the nominative singular.

#### Distribution

(Map 3).The species is known from a locality near the Bolivian border. 1 specimen examined.

**ARGENTINA**: SALTA: San Martín, Hito 1 (1).

#### Temporal distribution

.December (1).

#### Remarks.

This species is known only from the holotype. Like *Parochodaeus jujuyus*, it is from the far north of Argentina. It is probable that *Parochodaeus stupendus* will be discovered in Bolivia as well, especially because the name of the locality “Hito 1” is in reference to the “hito” or boundary post that indicates the border between Argentina and Bolivia.

### Gauchodaeus

Paulsen gen. n.

urn:lsid:zoobank.org:act:E0452D7A-435F-4AF6-A7A8-4CC28D1F4B80

http://species-id.net/wiki/Gauchodaeus

#### Type species.

*Gauchodaeus patagonicus* Paulsen & Ocampo, here designated.

#### Diagnosis.

The only known species in*Gauchodaeus* is an elongate ochodaeid with no elytral closing mechanism, and therefore can be quickly distinguished from the more globular *Parochodaeus* species with dentate elytral apices and a bituberculate propygidium. The genus is most similar to the genus *Synochodaeus* of southern Africa but is distinguished by the characters discussed above.

#### Description.

Ochodaeidae: Chaetocanthinae: Synochodaeini. Form convex, elongate. Sexual dimorphism lacking. Color testaceous to light reddish brown. ***Length:*** 5.7–5.9 mm. ***Width:*** 2.5–2.7 mm. ***Head:*** Surface densely tuberculate/punctate, setose. Mentum strongly tumid, not longitudinally impressed. Labrum densely punctate/setose. Distal 2 labial palpomeres spherical or irregularly shaped (not cylindrical). Clypeus long (length = ½ width), straight anteriorly, not reflexed. Frontoclypeal suture lacking distinct transverse sulcus. Mandibles relatively small, externally rounded, visible beyond labrum in dorsal view. Antenna with 10 antennomeres; 3-antennomere club oval, pubescent; first club antennomere neither strongly hemispherical nor enfolding distal antennomeres. Eyes large, somewhat bulging. ***Pronotum:*** Surface densely punctate, punctures moderate in size, with distinct anterior tubercle, setigerous; setae moderately long, testaceous. Margins beaded. ***Scutellum:*** Form weakly hastate with apex acute. ***Elytra:*** Striae (except sutural) not impressed, obsolete. Surface irregularly punctate; punctures moderate, each with small tubercle anteriorly, setigerous; setae dense, moderately long, testaceous. ***Legs*:** Protibia tridentate. Legs simple, unarmed. Mesotibial spur pectinate. Metatibia with large external carina in distal 1/3 long (extending > ½ width of tibia) but interrupted at middle. Metatibial spurs simple (possibly worn). Metatrochanter not acutely produced. ***Abdomen*:** Stridulatory peg absent. Propygidium short (length <1/5 width), without elytral locking mechanism.

#### Etymology.

The name is formed from *gaucho*, a local word commonly used to describe residents of the pampas or Patagonian grasslands, in combination with the root *Ochodaeus*. It is masculine in gender.

#### Composition.

Only one species is known, herein described.

#### Remarks.

 Nothing is known about the life history of these beetles, other than that they live in a rather inhospitable, arid part of Neuquén Province.

### Gauchodaeus patagonicus

Paulsen & Ocampo sp. n.

urn:lsid:zoobank.org:act:9904D088-2FE8-44A8-8691-0CE62AA2E36D

http://species-id.net/wiki/Gauchodaeus_patagonicus

Fig. 17 Map 4

#### Type material.

 Holotype male (IAZA), pinned. Original labels: a) “Piedra del Águila / (131) 525 m. / Neuquén – Argentina”; b) “7-II-92 / Leg. Mario Gentili”; c) on red paper “*Gauchodaeus / patagonicus* ♂ / Paulsen & Ocampo / HOLOTYPE”. Two paratypes (IAZA, MJPC) labeled as holotype. One paratype (CMNC) labeled: a) “Ea. Llamuco / (1100 M.S.N.M.) / Neuquén – Arg.”; b) “18-III-74 / LG. M. Gentili”; c) black-bordered “H. & A. Howden / Collection / ex. A. Martínez coll.”. One paratype (IAZA) labeled: “14-I-80 / Leg. M. Gentili”; “Bajada Marucho / (870 m.s.n.m.) / NEUQUÉN - ARG.” All paratypes labeled: “*Gauchodaeus / patagonicus* / Paulsen & Ocampo / PARATYPE” on yellow paper.

#### Type locality.

 Argentina: Neuquén: Piedra del Águila.

#### Diagnosis.

 The elongate body of this species (Fig. 17) is unlike that any other South American ochodaeids, which are in a different subfamily. The pectinate mesotibial spur, lack of propygidial tubercles, and simply rounded elytral apices will separate it from *Parochodaeus* or any other scarabaeoids.

#### Description, holotype male.

***Length:***5.2 mm. ***Width:***2.5 mm. ***Color:*** Reddish brown, shiny. ***Head:*** Surface tuberculate, punctate, with short setae increasing in length near eyes. Clypeus subtrapezoidal, long (length equal to 1/2 width). Mentum tumid. ***Pronotum:*** Surface punctate, tuberculate, tubercles small, setose. ***Elytra:*** Surface setose; setae moderately long, recumbent. ***Legs:*** Metafemur with posterior margin entire (not toothed in distal half). ***Abdomen:***Stridulatory peg absent.

**Description, paratypes** **(n=4). *Length:***5.7- 5.9 mm. ***Width:***2.5-2.7 mm. Specimens examined did not differ significantly from the holotype. The CMNC specimen lacks an abdomen.

#### Etymology.

The species is named for the region of Patagonia, where it is found. The name is used a masculine adjective in the nominative singular.

#### Distribution

**Map 4.**
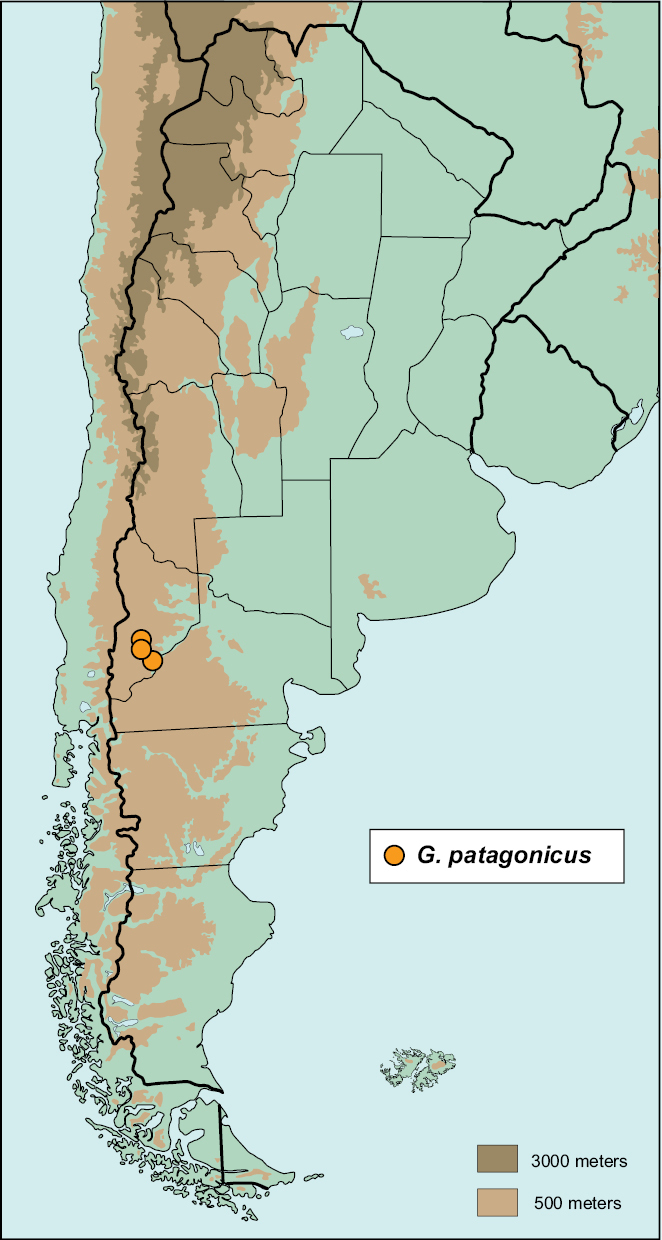
Distribution of *Gauchodaeus patagonicus* (circles)

(Map 4). **ARGENTINA:** NEUQUÉN: Bajada Marucho (1); Estancia Llamuco (1); Piedra del Águila (3).

#### Temporal fistribution.

January (1), February (3), March (1).

#### Remarks.

 This species is found in Neuquén province. All known specimens were collected by Mario Gentili in a desolate and arid habitat. The method of collection is not indicated on the labels, but it is probable that the species is nocturnally active and attracted to light.

## Supplementary Material

XML Treatment for Parochodaeus

XML Treatment for Parochodaeus campsognathus

XML Treatment for Parochodaeus cornutus

XML Treatment for Parochodaeus dentipes

XML Treatment for Parochodaeus jujuyus

XML Treatment for Parochodaeus perplexus

XML Treatment for Parochodaeus phoxus

XML Treatment for Parochodaeus proceripes

XML Treatment for Parochodaeus pudu

XML Treatment for Parochodaeus stupendus

XML Treatment for Gauchodaeus

XML Treatment for Gauchodaeus patagonicus
